# Inclinometer: A new device for measuring intermolar torque and angle

**DOI:** 10.34172/japid.2021.011

**Published:** 2021-07-13

**Authors:** Ahmad Behroozian, Les Kalman, Milad Hemmatiyan

**Affiliations:** ^1^Department of Orthodontics, Faculty of Dentistry, Tabriz University of Medical Sciences, Tabriz, Iran; ^2^Restorative Dentistry Chair, Dental Outreach, Schulich School of Medicine & Dentistry, Western University, Ontario, Canada; ^3^Architect, Private designer, Tabriz, Iran

**Keywords:** Appliance, measure, molar, torque

## Abstract

The torque of posterior teeth is of great importance in esthetics and occlusion. In the present article, we introduce a simple but useful device to measure intermolar torque. The device consists of two movable and adjustable arms that lie on the selected molar teeth bilaterally; the graduated plane at the body of the appliance then shows the intermolar torque. This device can measure intermolar torque easily and rapidly, with high validity and at a low cost.

## Introduction

The establishment of a proper torque is crucial in esthetics,^1^ periodontal health, and occlusion.^2^ It has been demonstrated that establishing an appropriate tooth position and its relationship with other teeth and surrounding structures is important to maintain a healthy periodontium.^3^ This is especially true in orthodontic tooth movement. During the expansion of the maxillary dental arch, the crown and root of the teeth can be moved buccally in different ratios and amounts. Expansion without attention to the inclination and torque of the teeth can result in periodontal breakdown.

Furthermore, improper torque of the posterior teeth increases the risk of traumatic occlusion for these teeth or even for the whole dentition. This, in turn, might jeopardize the periodontal health of the teeth and the integrity of the masticatory system. Some techniques are alleged to control the torque of posterior teeth, such as rectangular archwires.^4^ Some techniques have been introduced to measure intermolar torque during orthodontic treatment or in anthropometric studies. Linear gauge,^5^ CBCT images,^6^ protractors,^7^ and softwares^8^ have been proposed for intermolar torque measurement, but they have some disadvantages. The linear gauge cannot be used intraorally, and an additional calculation is needed. CBCT not only has a radiographic x-ray burden, but the accuracy of its measurement is under question. Therefore, we tried to introduce a simple and valid appliance to measure the torque of the molars relative to each other.

## Methods

**Figure 1:**
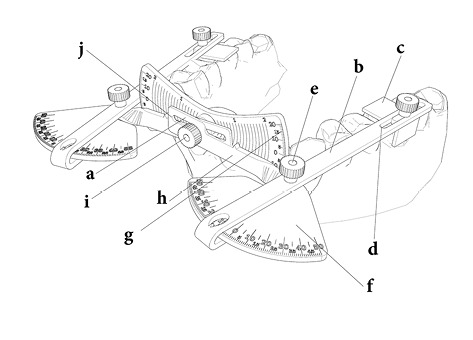
The components of the appliance.

The appliance consists of a body (a) and two movable arms (b) (Figure 1).

### Arms

An L-shaped plate (c) has been attached to the distal part of the arms, lying on the selected molar. The prepared hollow rail (d) ensures the possibility of mesiodistal movement of the L-shaped plate to adjust the location of the molars in large or small jaws and prepare the possibility of measuring the torque of first or second molars separately. This L-shaped plate has a positioning screw. One can move the L-shaped plate in the rail by loosening the screw and fix the plate by tightening it. The arms join the body with a vertical axis (e). This axis provides the possibility of accommodating the appliance with tapered, oval, or square arch forms. The horizontal graduated plane (f) shows an intermolar angle by rotating the arms around this vertical axis. The intermolar angle can be calculated by adding the left and right numbers of horizontal graduated planes.

### Body

**Figure 2:**
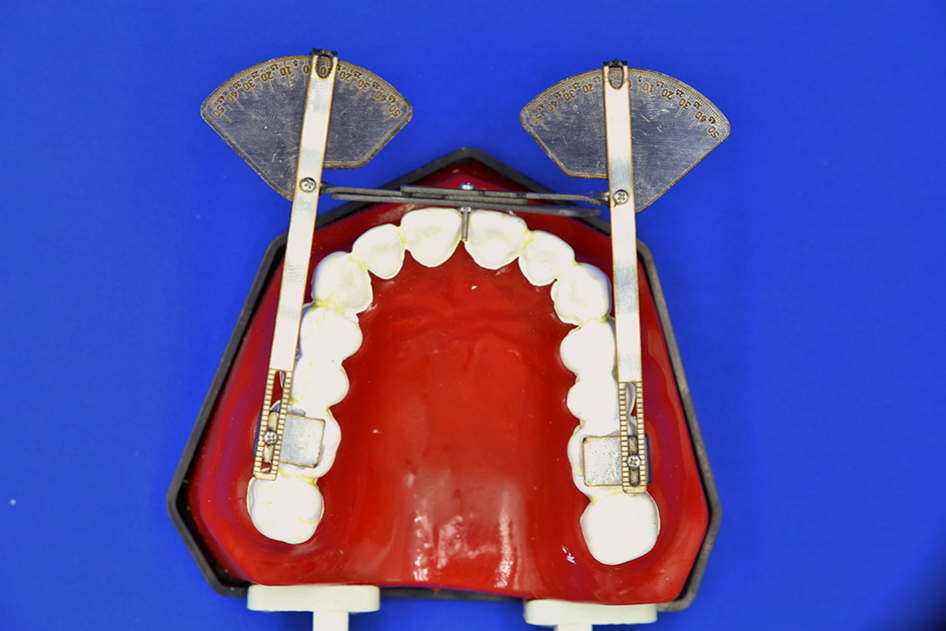
The inclinometer can be used on the stone models and in the oral cavity.

The body consists of a central graduated plate (g) and two bars (h) which join the central plane via a central axis (i). By placing the L-shaped plates on the molars, the bars rotate around the central axis, and intermolar torque can be calculated by adding the left and right numbers. The hollow rail (j) within the bars ensures the possibility of mediolateral movement to accommodate wide or narrow dental arches. These rails (d, j) and axes (e, i) make the appliance adjustable, and it can be used in a wide range of arch shapes and sizes. The appliance can measure intermolar torque both in the mouth and on the dental casts (Figure 2).

## Discussion

Some techniques have been introduced to measure the intermolar torque, but they have some disadvantages.

### Computed tomographic images (CT)

The CT technique for measuring intermolar torque has many disadvantages, including high cost, exposure to x-ray, and low validity.^9^ Coronal slices that can be used to measure intermolar angle are in a single plane, but sometimes the highest cusps of the target teeth on the left and right sides are not in the same plane. Therefore, the torque might be overestimated or underestimated. In addition, the coronal slice cannot be drawn parallel to the buccolingual plane of both the left and right molars because these molars have an offset degree to each other; therefore, it cannot reflect the torque correctly.^10^

### Linear gauge

This technique was introduced by Marshall et al,^5^ and it cannot be used intraorally, and dental stone models must be made. Intermolar torque is not measured directly, and a calculation is needed to determine it. Double measurements must be made, once for the left and once for the right side. The precision and reproducibility of the gauge are under question because the gauge must be in contact with three points, and any change in one of these single points can change the final score.

### Protractor

With the technique described by Jain et al, the adjustment read-out arm of the protractor lies on the buccal surfaces of the molars, and the scale of the protractor shows the molar torque.^7^ The major disadvantage of this technique is the false assumption of the buccal surface of the molar, which is not a flat surface; instead, it is convex. Therefore, the ruler cannot be properly adjusted on the buccal surface, and every examiner might place it differently. Also, the direction of the placement of the protractor and trimming of the cast base can influence the results.

Using digitalization with the help of software programs is not convenient. They are time-consuming and need specific landmarks.^8^ CBCT entails x-ray exposure and high costs to the patient.^6^ With these problems, it seems that they cannot be used for routine practice, and there is some skepticism over their validity.

### Limitations and suggestions

Inter- and intra-examiner repeatability of the appliance must be calculated both on the cast and in theoral cavity.

## Conclusions

The inclinometer provides an easy, fast, and valid measurement of intermolar torque. It can be used intraorally or on the casts. In addition, it can be used to assess treatment progress and compare the effectiveness of different appliances in the application and control of root torques.

## Authors’ contributions

AB initiated, conceptualized, and supervised the research work. MN and AB made the appliance. AB and LK prepared and critically revised the manuscript. All authors have contributed to analyzing the data and writing the manuscript.

## Ethics approval

Not applicable.

## Competing interests

The authors declare no conflicts of interest.

## References

[1] Zachrisson BU (2006). Buccal uprighting of canines and premolars for improved smile esthetics and stability. World J Orthod.

[2] American Board of Orthodontics. Grading system for dental casts and panoramic radiographs. St. Louis: American Board of Orthodontics; 1999. 10.1016/s0889-5406(98)70179-9 9810056

[3] Morris JW, Campbell PM, Tadlock LP, Boley J, Buschang PH (2017). Prevalence of gingival recession after orthodontic tooth movements. Am J Orthod Dentofacial Orthop.

[4] Papageorgiou SN, Sifakakis I, Doulis I, Eliades T, Bourauel C (2016). Torque efficiency of square and rectangular archwires into 0018 and 0022 in conventional brackets. Prog Orthod.

[5] Marshall S, Dawson D, Southard KA, Lee AN, Casko JS, Southard TE (2003). Transverse molar movements during growth. Am J Orthod Dentofacial Orthop.

[6] Tong H, Kwon D, Shi J, Sakai N, Enciso R, Sameshima GT (2012). Mesiodistal angulation and faciolingual inclination of each whole tooth in 3-dimensional space in patients with near-normal occlusion. Am J Orthod Dentofacial Orthop.

[7] Jain S, Kiran H J, Neha K, Bhattacharjee D, Rana S, Nayyar AS (2017). Assessment of tip, torque, and tooth size discrepancies in Angle’s class II division 2 malocclusion. Int J Orofac Biol.

[8] Talaat S, Kaboudan A, Breuning H, Ragy N, Elshebiny T, Kula K, Ghoneimag A (2015). Reliability of linear and angular dental measurements with the OrthoMechanics Sequential Analyzer. Am J Orthod Dentofacial Orthop.

[9] Raman SP, Mahesh M, Blasko RV, Fishman EK (2013). CT scan parameters and radiation dose: practical advice for radiologists. J Am Coll Radiol.

[10] Cavaignac E, Lecoq M, Ponsot A, Moine A, Bonnevialle N, Mansat P, Sans N, Bonnevialle P (2013). CT scan does not improve the reproducibility of trochanteric fracture classification: a prospective observational study of 53 cases. Orthop Traumatol Surg Res.

